# The fission yeast NDR kinase Orb6 and its signalling pathway MOR regulate cytoplasmic microtubule organization during the cell cycle

**DOI:** 10.1098/rsob.230440

**Published:** 2024-03-06

**Authors:** Kazunori Kume, Kenji Nishikawa, Rikuto Furuyama, Takahiro Fujimoto, Takayuki Koyano, Makoto Matsuyama, Masaki Mizunuma, Dai Hirata

**Affiliations:** ^1^ Graduate School of Integrated Sciences for Life, Hiroshima University, 1-3-1 Kagamiyama, Higashi-Hiroshima, Hiroshima 739-8530, Japan; ^2^ Hiroshima Research Center for Healthy Aging (HiHA), Hiroshima University, Higashi-Hiroshima, Hiroshima 739-8530, Japan; ^3^ Division of Cell Biology, Shigei Medical Research Institute, 2117 Yamada, Minami-ku, Okayama 701-0202, Japan; ^4^ Division of Molecular Genetics, Shigei Medical Research Institute, 2117 Yamada, Minami-ku, Okayama 701-0202, Japan; ^5^ Faculty of Agriculture, Niigata University, 2-8050 Ikarashi, Niigata 950-2181, Japan; ^6^ Sakeology Center, Niigata University, 2-8050 Ikarashi, Niigata 950-2181, Japan

**Keywords:** fission yeast, microtubule organization, NDR kinase, cell cycle

## Abstract

Microtubule organization and reorganization during the cell cycle are achieved by regulation of the number, distribution and activity of microtubule-organizing centres (MTOCs). In fission yeast, the Mto1/2 complex determines the activity and distribution of cytoplasmic MTOCs. Upon mitosis, cytoplasmic microtubule nucleation ceases; inactivation of the Mto1/2 complex is triggered by Mto2 hyperphosphorylation. However, the protein kinase(s) that phosphorylates Mto2 remains elusive. Here we show that a conserved signalling network, called MOR (morphogenesis Orb6 network) in fission yeast, negatively regulates cytoplasmic MTOCs through Mto2 phosphorylation to ensure proper microtubule organization. Inactivation of Orb6 kinase, the most downstream MOR component, by attenuation of MOR signalling leads to reduced Mto2 phosphorylation, coincident with increased number of both Mto2 puncta and cytoplasmic microtubules. These defects cause the emergence of uncoordinated mitotic cells with cytoplasmic microtubules, resulting in reduced spindle assembly. Thus, the regulation of Mto2 by the MOR is crucial for cytoplasmic microtubule organization and contributes to reorganization of the microtubule cytoskeletons during the cell cycle.

## Graphical abstract

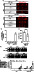

## 1. Introduction

Microtubules are essential for many cellular processes, such as intracellular transport of proteins and organelles, establishment of cell polarity, cell motility and chromosome segregation [1–5]. In order to facilitate these different processes at appropriate times during the cell cycle and cell differentiation, microtubules are organized into specific arrangements by microtubule-organizing centres (MTOCs) [2,4,5]. For instance, in most eukaryotic cells, the MTOC(s) forms polarized microtubule arrays during interphase and these arrays undergo reorganization to form the bipolar spindle in mitosis. This reorganization proceeds rapidly and precisely during the transition between interphase and mitosis and vice versa. However, the regulation mechanisms coupling cytoskeletal changes with the cell cycle are poorly understood.

The fission yeast *Schizosaccharomyces pombe* is an excellent model system in which to study cell cycle-dependent microtubule organization by distinct types of MTOCs: the centrosomal MTOC (spindle pole body, SPB) and non-centrosomal MTOCs (interphase MTOC: iMTOC; and equatorial MTOC: eMTOC) [6,7]. During interphase, cytoplasmic microtubules are nucleated from the cytoplasmic face of the SPB, nuclear membrane bound iMTOCs, and pre-existing microtubules to organize a longitudinal array of two to five microtubule bundles [6,7]. During the transition period from interphase to mitosis, nucleation of cytoplasmic microtubules from the iMTOCs ceases and simultaneously intranuclear mitotic spindle microtubules start nucleating from the nucleoplasmic face of the SPB [6,7]. During anaphase, astral microtubules are nucleated from the cytoplasmic face of the SPBs, and then at the end of mitosis, cytoplasmic microtubules are nucleated from the eMTOC localized to the contractile actomyosin ring, to form a post-anaphase array [6,7].

Nucleation of the cytoplasmic microtubules is dependent on the Mto1/2 complex, which is composed of Mto1 and Mto2 proteins and localizes to the cytoplasmic MTOCs during interphase [8–14]. This complex interacts with the γ-tubulin complex and recruits it to the cytoplasmic MTOCs [8–14]. Upon mitotic entry, the Mto1/2 complex is inactivated and disassembled by phosphorylation of Mto2 at multiple sites [14]. However, which kinase phosphorylates Mto2 and how its regulation integrates into cell cycle coupled organization and reorganization of the microtubule cytoskeleton remain elusive.

A conserved signalling network, the MOR (morphogenesis Orb6 network) [15–17] is essential for the establishment of cell polarity following cytokinesis and the control of actin-based polarized growth during interphase [16], in part through spatial regulation of the GTPase Cdc42 [18]. Furthermore, the MOR is important for actin reorganization during the transition from mitosis to interphase, cross talking with the SIN signalling pathway, which is essential for actomyosin constriction, and septum formation during cytokinesis [19]. The MOR pathway is composed of conserved proteins including Pmo25 (the MO25 family protein [16,20–23]) and Nak1 (a GC kinase [16,24,25]), all of which act upstream of Mor2 (the *Drosophila* Furry/Fry protein [15,16,26]) and Orb6 (the NDR kinase [16,17,27,28]), downstream components of the pathway. Orb6 kinase activity oscillates during the cell cycle: it is activated during polarized growth and inactivated during mitosis and its kinase activity is dependent on its interacting protein Mor2 and upstream regulators Pmo25 and Nak1. Interestingly, study of mammalian Fry and NDR kinase revealed that these proteins are involved in mitotic spindle organization and chromosome alignment [29–32], suggesting that Fry and NDR kinase regulate not only actin but also microtubule cytoskeletons in some species and cell types. However, physiological substrates of NDR kinase and the roles of Fry and NDR kinase in microtubule organization and reorganization during the cell cycle remain elusive.

In this study, we show that the MOR negatively regulates non-centrosomal MTOCs, by phosphorylation of Mto2, to ensure proper organization of cytoplasmic microtubules during polarized growth in interphase. Our findings also suggest that the regulation of Mto2 by the MOR is crucial for microtubule reorganization during the transition from interphase to mitosis to ensure mitotic spindle assembly.

## 2. Results

### 2.1. Microtubule reorganization during the transition from interphase to mitosis is impaired in MOR mutants

**Figure 1.**
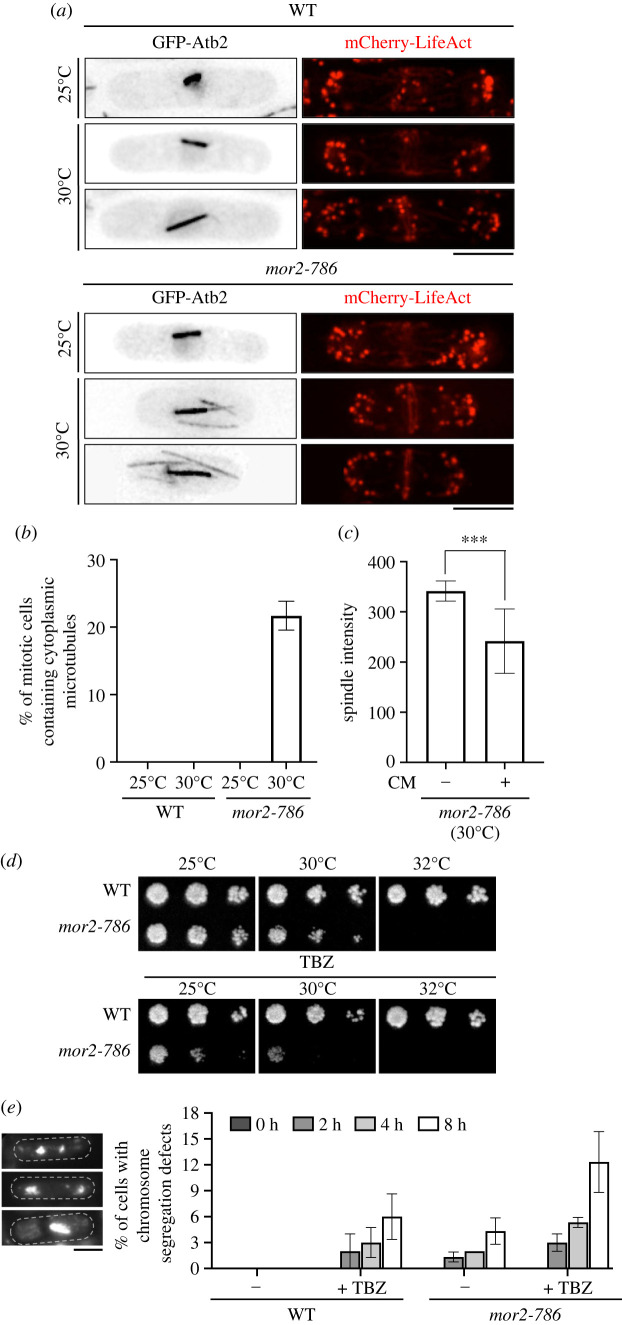
Mutation of the MOR causes the emergence of abnormal mitotic cells containing interphase cytoplasmic microtubules and increases the risk of chromosome segregation defects. (*a*) GFP-Atb2 (microtubules) and mCherry-LifeAct (actin) of mitotic cells in WT and *mor2-786* mutant strains (25°C and 30°C for 2 h). Scale bar, 5 µm. (*b*) Frequency of abnormal mitotic cells containing cytoplasmic microtubules in WT and *mor2-786* mutant strains (25°C and 30°C for 2 h) (*n* = 100). Cells with spindle lengths between 1 and 4 µm measured. (*c*) Fluorescent signal intensities of GFP-Atb2 (spindle microtubule) in *mor2-786* mutant cells with (+) or without (−) cytoplasmic microtubules (CM) (*n* > 20). ***p < 0.001 (Mann–Whitney test, two-tailed). (*d*) Growth properties of WT and *mor2-786* mutant strains on YE4S or YE4S containing microtubule destabilizing drug TBZ (13 µg ml^−1^) at indicated temperatures (25, 30 and 32°C). (*e*) Left panel: representative DAPI-stained images of the cells with defects in mitotic chromosome segregation. Scale bar, 5 µm. Right panel: the percentage of cells with abnormal mitotic chromosome segregation in WT and *mor2-786* mutant strains untreated or treated with TBZ (18 µg ml^−1^). Exponential growth cells grown at 25°C were shifted up at 30°C and incubated for 8 h with or without TBZ. Cells were fixed with formaldehyde at indicated time points (0, 2, 4 and 8 h) and stained with DAPI.

We have previously shown that loss of Orb6 kinase activity in MOR mutants disrupts polarized actin organization resulting in round morphology and cessation of growth in G2 phase at the restrictive temperature [15,16,27]. To explore the roles of the MOR in the microtubule cytoskeleton of growing cells, we compared the microtubule structure of wild-type cells with that of temperature-sensitive *mor2-786* mutant cells, a representative mutant of the MOR [15,16,27], grown at semi-restrictive temperature (30°C), by imaging cells expressing GFP-tagged Atb2, one of two alpha tubulin genes in fission yeast [33]. We first examined mitotic cells in which spindle microtubules and/or actomyosin ring were formed (figure 1*a*) and found that in cells with short spindles (metaphase spindle length 1–4 µm), approximately 20% of *mor2-786* mutant cells contained one or more cytoplasmic microtubules, whereas such microtubules were not observed in wild-type cells (figure 1*a*,*b*). This result indicates that reorganization of the microtubule cytoskeleton from interphase to mitosis is impaired by mutation of the MOR. Since cytoplasmic and spindle microtubules share same microtubule elements such as α/β-tubulins, the presence of cytoplasmic microtubules in mitotic cells might affect spindle assembly. To test this possibility, we quantified the intensity of spindle microtubules (GFP-Atb2) in *mor2-786* mutant cells that do or do not contain cytoplasmic microtubules. As expected, the intensity of spindle microtubules in *mor2-786* mutant cells with cytoplasmic microtubules was significantly reduced compared to that in *mor2-786* mutant cells without cytoplasmic microtubules (figure 1*c*), indicating that the existence of cytoplasmic microtubules in mitotic cells attenuates their spindle assembly.

A reduction in spindle assembly could increase the risk of chromosome segregation defects. To investigate this, we examined the growth of *mor2-786* mutant cells on YE4S (rich) agar plates containing the microtubule-depolymerizing drug TBZ. At 25°C, the *mor2-786* mutant strain was sensitive to TBZ, at 30°C this sensitivity was enhanced (figure 1*d*) and the frequency of mitotic cells containing cytoplasmic microtubules was increased (figure 1*b*). Furthermore, compared to wild-type cells, the percentage of cells with defects in mitotic chromosome segregation was increased in *mor2-786* mutant cells grown in YE4S liquid culture at 30°C in the presence or even absence of TBZ (figure 1*e*). We confirmed that other MOR mutants were also sensitive to TBZ at the permissive temperature (electronic supplementary material, figure S1*a*). These results suggest that the MOR is important for proper microtubule reorganization during the transition from interphase to mitosis, and that the completion of this reorganization is necessary for spindle assembly, which is critical for proper chromosome segregation.

### 2.2. The number of cytoplasmic microtubules in interphase is increased in MOR mutants

**Figure 2.**
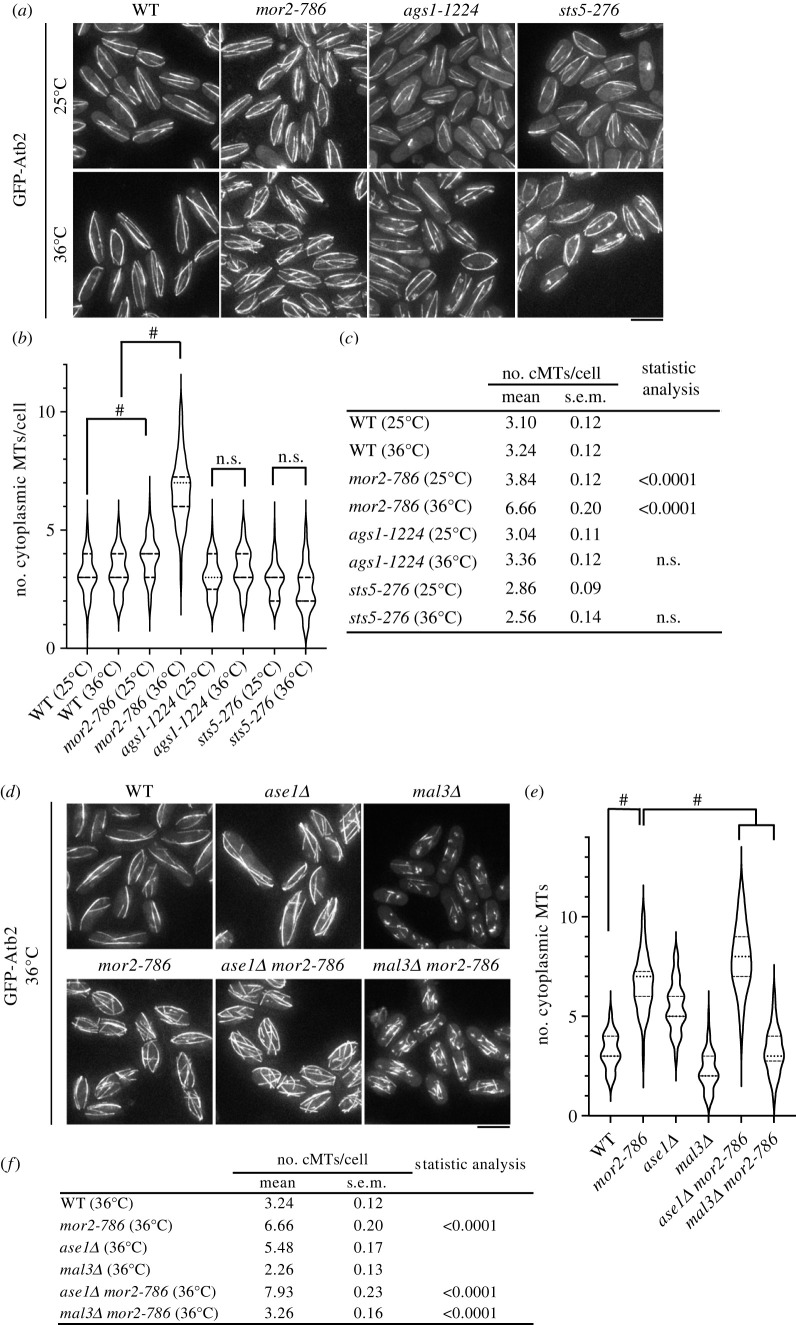
A defect in the MOR causes an increased number of cytoplasmic microtubules. (*a*) GFP-Atb2 (microtubules) of wild-type, *mor2-786*, *ags1-1224* and *sts5-276* cells (25°C or 36°C, 2 h). Scale bar, 5 µm. (*b*) The number of interphase cytoplasmic microtubules per cell in the indicated strains (*n* > 50) is presented as a violin plot. Dashed lines and dotted lines indicate quartiles and median, respectively. (*c*) Summary table of data in (*b*). cMTs, cytoplasmic microtubules. ^#^*p <* 0.0001 (Mann–Whitney test, two-tailed). (*d*) GFP-Atb2 (microtubules) of wild-type, *mor2-786*, *ase1Δ*, *mal3Δ*, *ase1Δmor2-786* and *mal3Δmor2-786* cells (36°C, 2 h). Scale bar, 5 µm. (*e*) The number of interphase cytoplasmic microtubules per cell in the indicated strains (*n* > 50) is presented as a violin plot. Dashed lines and dotted lines indicate quartiles and median, respectively. (*f*) Summary table of data in (*e*). ^#^*p <* 0.0001 (Mann–Whitney test, two-tailed).

We next investigated the organization of interphase cytoplasmic microtubules. Wild-type cells have between two and five cytoplasmic microtubule bundles that are oriented along the long axis of the cell at the permissive, semi-restrictive and restrictive temperatures (figure 2*a–c*; electronic supplementary material, figure S1*b*–*d*). In *mor2-786* mutant cells at 25°C, the average number of cytoplasmic microtubules was slightly higher than that in wild-type cells and after incubation at 30°C for 2 h, *mor2-786* mutant cells displayed an increased average number of cytoplasmic microtubules (with the number ranging from 2 to 7) (figure 2*a–c*; electronic supplementary material, figure S1*c*,*d*). This phenotype was enhanced at the restrictive temperature, *mor2-786* mutant cells displayed between 3 and 10 cytoplasmic microtubules at 36°C (figure 2*a–c*) and was also similarly observed in other MOR mutants (*pmo25-35*, *nak1-125* and *orb6-25* mutant strains) and a constitutive active SIN mutant (*cdc3 cdc16*) [19], in which Orb6 kinase activity is inhibited (electronic supplementary material, figures S1*e*,*f* and S2). These results indicate that the MOR is required for proper number of cytoplasmic microtubules. We next investigated whether the increase in number of cytoplasmic microtubules observed in the MOR mutants is due to their round morphology using other morphological round mutants: cell wall synthesis defective mutant *ags1-1224* [34] and mRNA degradation defect mutant *sts5-276* [35,36]. The number of cytoplasmic microtubules in these round mutant cells was similar to that in wild-type cells at 36°C (figure 2*a–c*), suggesting that the increased number of cytoplasmic microtubules observed in the MOR mutants is not a result of the round morphology and may be a specific phenotype of the MOR mutants. Taken together, the MOR is required for proper organization of interphase cytoplasmic microtubules.

There are at least two possible causes of the increase in number of cytoplasmic microtubules: one is a microtubule-bundling defect and the other is an increased number of microtubule nucleation sites. To distinguish between these possibilities, we constructed a double mutant of *mor2-786* and deletion of *ase1* [37,38]*,* which encodes a microtubule-bundling factor, and compared the cytoplasmic microtubules of double-mutant cells to that of single-mutant cells. *ase1Δ* cells are defective in forming microtubule-overlapping regions [37,38], resulting in an approximate doubling of the number of cytoplasmic microtubules compared to wild-type cells (figure 2*d–f*). Although the average number of cytoplasmic microtubules in *ase1Δ* cells is higher than that in WT cells at 36°C (figure 2*e*,*f*), the higher number of cytoplasmic microtubules in the *ase1Δ mor2-786* double mutant than in either single mutant (figure 2*e*,*f*). This result indicates that the increase in number of cytoplasmic microtubules in *mor2-786* mutant cells might be caused by an increased number of microtubule nucleation sites rather than microtubule-bundling defects. Indeed, when cytoplasmic microtubules were shortened in *mor2-786* mutant cells by deletion of *mal3* [39], a microtubule-plus end binding protein and fission yeast EB1 homologue, the double-mutant cells had more microtubule stubs than *mal3Δ* mutant cells and the number of stubs was similar to that in *mor2-786* single-mutant cells at 36°C (figure 2*d–f*).

### 2.3. The cytoplasmic microtubule number increase induced by the MOR mutation is dependent on the Mto1/2 complex

**Figure 3.**
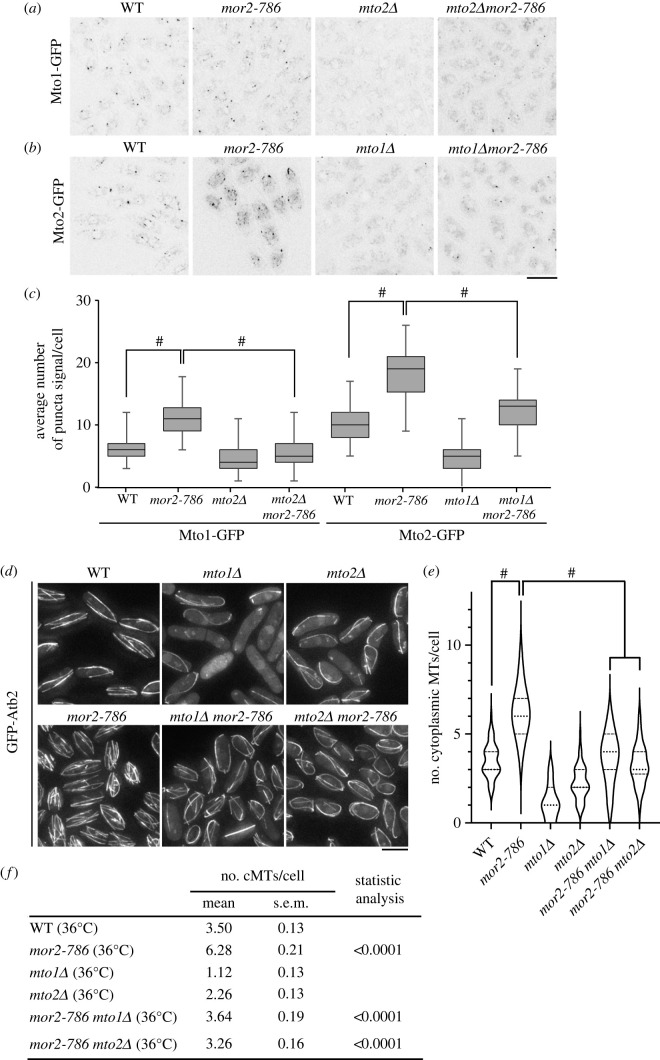
Increased number of cytoplasmic microtubules induced by a MOR defect is dependent on Mto1 and Mto2. (*a*) Localization of Mto1-GFP in wild-type, *mor2-786*, *mto2Δ* and *mto2Δmor2-786* cells (36°C, 2 h). Scale bar, 5 µm. (*b*) Localization of Mto2-GFP in wild-type, *mor2-786*, *mto1Δ* and *mto1Δmor2-786* cells (36°C, 2 h). Scale bar, 5 µm. (*c*) Number of fluorescent signals of Mto1-GFP or Mto2-GFP puncta per cell in the indicated strains (36°C, 2 h) (*n* > 50) is shown as a box and whiskers plot. ^#^*p <* 0.0001 (*d*) GFP-Atb2 (microtubules) of wild-type, *mto1Δ*, *mto2Δ*, *mor2-786*, *mto1Δmor2-786*, and *mto2Δmor2-786* cells (36°C, 2 h). Scale bar, 5 µm. (*e*) Number of cytoplasmic microtubules per cell in the indicated strains (36°C for 2 h) is presented as a violin plot. Dashed lines and dotted lines indicate quartiles and median, respectively. (*f*) Summary table of data in (*e*). ^#^*p <* 0.0001 (Mann–Whitney test, two-tailed).

To confirm that the number of microtubule nucleation sites is increased in the MOR mutants, we examined the localization of the Mto1/2 complex [8–12], which determines all cytoplasmic MTOC sites, in wild-type and *mor2-786* mutant cells grown at both permissive and restrictive temperatures. At 25°C, the average number of Mto1-GFP and Mto2-GFP puncta in *mor2-786* mutant cells was almost equivalent to that in wild-type cells (electronic supplementary material, figure S3*a*). Whereas, following incubation at 36°C for 2 h, the average number of Mto1-GFP and Mto2-GFP puncta in *mor2-786* mutant cells was approximately twice that in wild-type cells (figure 3*a*–*c*) but their fluorescent intensities at iMTOCs (non-centrosomal MTOCs) and their protein levels in *mor2-786* mutant cells at 36°C were almost equivalent to those in wild-type cells (electronic supplementary material, figure S3*b–e*). These results indicate that the number of cytoplasmic microtubule nucleation sites is increased but the protein level of Mto1 and Mto2 is not altered in *mor2-786* mutant cells.

Mto1 and Mto2 are mutually dependent for formation of their puncta and proper localization (figure 3*a–c*) [9,10]. To investigate this in the *mor2-786* mutant background, we examined localization of Mto1-GFP and Mto2-GFP in *mto2Δ mor2-786* and *mto1Δ mor2-786* double-mutant cells respectively*.* The increased average number of Mto1-GFP or Mto2-GFP puncta observed in *mor2-786* mutant cells at 36°C was reduced by deletion of *mto2* or *mto1* (figure 3*a–c*), indicating that the Mto1/2 complex is required for the increased number of cytoplasmic microtubule nucleation sites in *mor2-786* mutant cells.

To determine whether the increased number of cytoplasmic microtubules observed in *mor2-786* mutant cells is dependent on the Mto1/2 complex, we used the deletion mutants of *mto1* and *mto2*, which are both defective in nucleation of cytoplasmic microtubules [8–10]. Deletion of either *mto1* or *mto2* suppressed the increase in number of cytoplasmic microtubules observed in *mor2-786* mutant cells (figure 3*d*–*f*). We also confirmed that deletion of either *mto1* or *mto2* suppressed the increase in number of cytoplasmic microtubules observed in other MOR mutants (electronic supplementary material, figure S4). Taken together, these results indicate that Mto1 and Mto2 are required for the increase in number of cytoplasmic microtubules induced by the MOR mutations.

### 2.4. The MOR is involved in Mto2 phosphorylation during polarized growth

**Figure 4.**
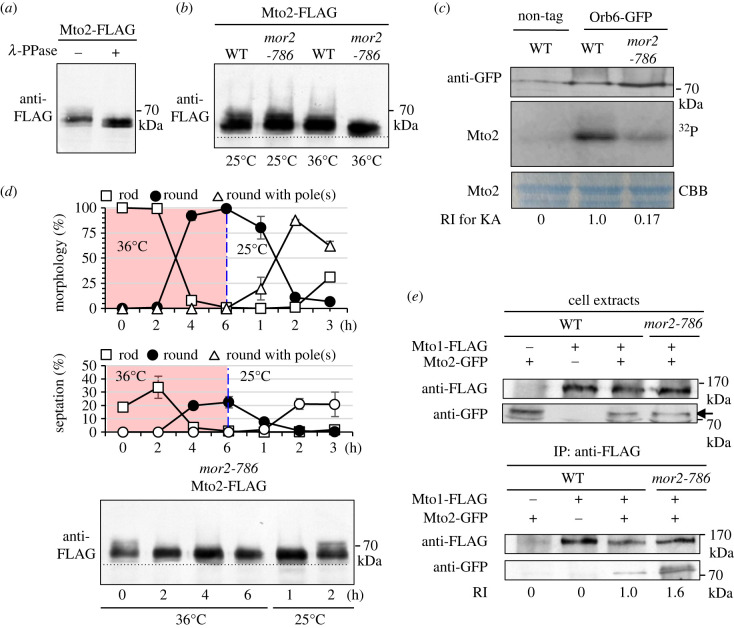
The MOR is involved in phosphorylation of Mto2 during polarized growth. (*a*) Phosphatase treatment of Mto2. Proteins in cell extracts from wild-type cells expressing Mto2-FLAG grown at 25°C were separated by SDS-PAGE. Treatment with λ-protein phosphatase was conducted before electrophoresis. Mto2-FLAG was detected by immunoblotting with anti-FLAG antibody. (*b*) Phosphorylation level of Mto2 in wild-type and *mor2-786* mutant strains. Cells expressing Mto2-FLAG were grown at 25°C or were grown at 25°C then shifted to 36°C and incubated for 2 h. Proteins in cell extracts were separated by SDS-PAGE. (*c*) An *in vitro* kinase assay for Mto2 protein mixed with Orb6-GFP from wild-type or *mor2-786* mutant cells grown at 36°C for 2 h. Relative intensity (RI) of the kinase activity (KA) was evaluated by ^32^P signal intensity from the middle lane of Mto2 protein mixed with immunoprecipitated Orb6-GFP from wild-type cells. (*d*) Cell morphology and phosphorylation level of Mto2 in the *mor2-786* mutant strain. Cells expressing Mto2-FLAG were grown at 25°C, shifted to 36°C and incubated for 6 hours, and then shifted down to 25°C and incubated for 3 hours. Cells were fixed with formaldehyde and stained with calcofluor to monitor the growth polarity (*n* > 200). (*e*) Immunoprecipitation between Mto1-FLAG and Mto2-GFP in wild-type and *mor2-786* strains (36°C, 2 h). Cell extracts were immunoprecipitated with anti-FLAG antibody, and the precipitated proteins (IP) were detected by immunoblotting with anti-FLAG antibody or anti-GFP antibody. Relative intensity (RI) of the interaction was evaluated by the amount of the co-immunoprecipitated Mto2-GFP protein from wild-type cell extract.

Cytoplasmic microtubule nucleation and organization in fission yeast are coordinated with the cell cycle and their regulation during mitosis is dependent on the phosphorylation level of Mto2 [14]: when cells enter mitosis, Mto2 is hyperphosphorylated coincidentally with disassembly of the Mto1/2 complex and cytoplasmic microtubule nucleation ceases. A non-phosphorylatable *mto2* mutation increases the number of cytoplasmic microtubules in interphase and retains cytoplasmic microtubules in mitosis [14]. This microtubule phenotype of the *mto2* non-phosphorylatable mutant resembles that in the MOR mutants, indicating that phosphorylation level of Mto2 might be decreased in the MOR mutant cells. To investigate this possibility, we examined the phosphorylation level of Mto2 in wild-type and *mor2-786* mutant cells. We confirmed that Mto2 protein was phosphorylated in asynchronous wild-type cells (figure 4*a*). Although at 25°C, the migration patterns of Mto2 protein in wild-type and *mor2-786* mutant cells were similar, after incubation at 36°C for 2 h, the amount of phosphorylated Mto2 in *mor2-786* mutant cells was decreased, compared to that in wild-type cells and the fast-migrating lower band migrated faster than that in wild-type cells (figure 4*b*). This result indicates that the MOR is involved in phosphorylation of Mto2. To confirm that Orb6 phosphorylates Mto2, we performed an *in vitro* kinase assay using immunoprecipitated Orb6-GFP from wild-type or *mor2-786* mutant cells grown at the restrictive temperature and purified Mto2 from bacteria. We found that Orb6-GFP immunoprecipitated from wild-type cells phosphorylated Mto2 (figure 4*c*), while Orb6-GFP immunoprecipitated from *mor2-786* mutant cells almost abolished phosphorylation, indicating that Orb6 phosphorylates Mto2 directly or indirectly.

We next investigated when phosphorylation of Mto2 by the MOR occurs during the cell cycle, using *cdc10-129* and *cdc25-22* mutants that block cell cycle progression at G1 and G2 phases, respectively [40,41]. Although at 36°C, the migration patterns of Mto2 protein in G1 arrested *cdc10-129* mutant and asynchronous wild-type cells were similar, the amount of phosphorylated Mto2 in G2 arrested *cdc25-22* mutant cells was increased, compared to that in wild-type cells and the slow-migrating lower band migrated slower than that in wild-type cells (electronic supplementary material, figure S5), indicating that Mto2 is more phosphorylated in G2 phase. In the double mutants of *mor2-786 cdc10-129* and *mor2-786 cdc25-22* at 36°C, the migration patterns of Mto2 protein were similar to that in *mor2-786* mutant cells, indicating that the MOR may be involved in Mto2 phosphorylation in interphase. To investigate whether the Mto2 phosphorylation by the MOR occurs during polarized growth in interphase, we used temperature-dependent reversible phenotype of the *mor2-786* mutant cells [15]. The mutant cells grown at 25°C were shifted up to 36°C, incubated for 6 h, and then shifted down to 25°C and incubated for 3 h (figure 4*d*). The phosphorylation level of Mto2 was decreased at 36°C after 2 h and this level was maintained following incubation at 36°C for 6 h, during which most cells became spherical in morphology and were unseptated (figure 4*d*). After shift down to 25°C and incubation for 2 h, the round cells re-established cell polarity and started polarized growth without septation. During this morphological change, Mto2 was phosphorylated again, and the phosphorylation level returned to its former state observed in the *mor2-786* mutant cells grown at 25°C (figure 4*d*), indicating that the MOR-dependent phosphorylation of Mto2 occurs during polarized growth in interphase.

Phosphorylation of Mto2 inhibits the interaction between Mto1 and Mto2 [14]. To investigate whether the reduction in phosphorylation level of Mto2 in the MOR mutants increases the interaction between Mto1 and Mto2, we examined the physical interaction of these proteins in wild-type cells and *mor2-786* mutant cells at 36°C. The amount of Mto1-associated Mto2 protein was increased 1.6-fold in *mor2-786* mutant cells compared with that in wild-type cells (figure 4*e*), indicating that the reduction in the phosphorylation level of Mto2 by MOR mutation enhances the physical interaction between Mto1 and Mto2 and this may cause the increased number of Mto1 and Mto2 puncta, resulting in an increased number of cytoplasmic microtubules during interphase.

### 2.5. Recruitment of Orb6 kinase with Mto2 phosphorylates Mto2 and inhibits formation of Mto2 puncta and cytoplasmic microtubules

**Figure 5.**
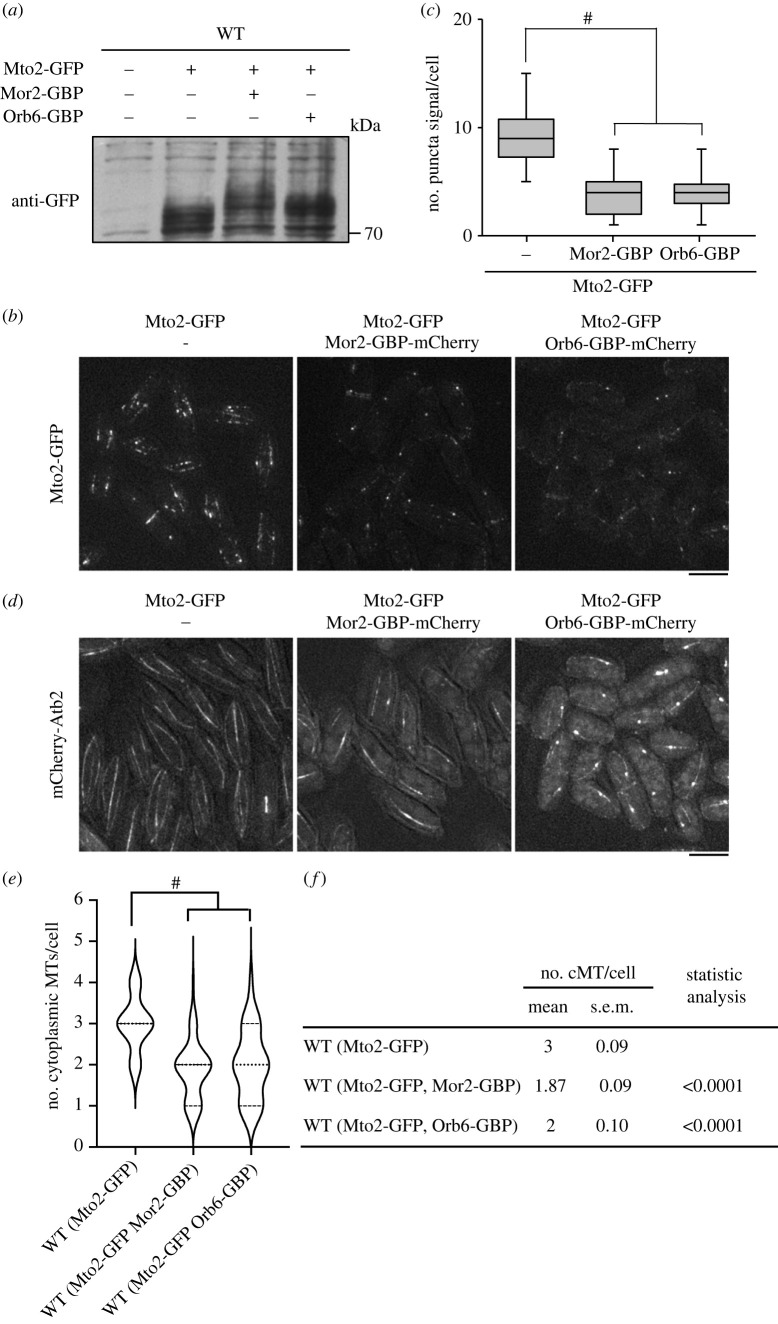
Forced association of Mor2 or Orb6 to Mto2 negatively regulates the function of Mto2 by its phosphorylation. (*a*) Phosphorylation level of Mto2 in cells expressing Mto2-GFP, Mto2-GFP and Mor2-GBP-mCherry, or Mto2-GFP and Orb6-GBP-mCherry. Proteins in cell extracts from cells grown at 25°C were separated by SDS-PAGE. Mto2-GFP was detected by immunoblotting with anti-GFP antibody. (*b*) Localization of Mto2-GFP in the indicated strains (25°C). Scale bar, 5 µm. ^#^*p <* 0.0001 (Mann–Whitney test, two-tailed). (*c*) Number of fluorescent signals of Mto2-GFP puncta per cell in the indicated strains (25°C) (*n* > 50) is shown as a box and whiskers plot. (*d*) GFP-Atb2 (microtubules) of the indicated strains (25°C). Scale bar, 5 µm. (*e*) The number of interphase cytoplasmic microtubules per cell in the indicated strains (*n* > 50) is presented as a violin plot. Dashed lines and dotted lines indicate quartiles and median, respectively. (*f*) Summary table of data in (*e*). ^#^*p <* 0.0001 (Mann–Whitney test, two-tailed).

We next investigated whether Mto2 phosphorylation by the MOR, especially the most downstream kinase Orb6, inhibits the formation of Mto2 puncta and nucleation of cytoplasmic microtubules in a wild-type background. To test this, we used the GFP-binding protein (GBP) [42] (tagged with mCherry, GBP-mCherry) fused to the C-terminus of endogenous Orb6 or its interacting protein Mor2 to bind to GFP-tagged Mto2. Mor2 and Orb6 are localized to both cell ends during interphase [16], but when cells expressing both Mto2-GFP and Mor2-GBP-mCherry or Orb6-GBP-mCherry, Mor2-GBP-mCherry or Orb6-GBP-mCherry co-localized with Mto2-GFP (electronic supplementary material, figure S6). In these co-expressed situations, Mto2 was more highly phosphorylated (figure 5*a*) than control strain or strains co-expressing unrelated kinases tagged with GBP (figure 5*a*; electronic supplementary material, figure S7), but the phosphorylation level of Mto2 in the cells co-expressing with Mor2-GBP-mCherry is higher than that in the cells co-expressing with Orb6-GBP-mCherry. This result suggests that in addition to Orb6 kinase, other protein kinases interacted with Mor2 and were involved in Mto2 phosphorylation when Mor2 bound to Mto2.

Remarkably, the cells co-expressing Mto2-GFP and Mor2-GBP-mCherry or Orb6-GBP-mCherry displayed almost diminished Mto2-GFP puncta except for one dot (figure 5*b*,*c*), which might be the SPB, and the number of cytoplasmic microtubules was drastically reduced compared with that in control cells expressing only Mto2-GFP (figure 5*d–f*). This microtubule phenotype was almost equivalent to that in *mto2Δ* cells (figure 3*d–f*), indicating that the MOR has a negative role in nucleation of cytoplasmic microtubules through inhibition of Mto2 by its phosphorylation.

## 3. Discussion

Here, we report a novel function of a conserved signalling network of fission yeast, called MOR, in regulation of cytoplasmic MTOCs to organize interphase cytoplasmic microtubules. This regulation is important for microtubule reorganization during the transition from interphase to mitosis. Our data show that the MOR is involved in phosphorylation of Mto2 during polarized growth and negatively regulates it for proper organization of cytoplasmic microtubules. Loss of function of the MOR in a *mor2-786* mutant in which Orb6 kinase activity is decreased [16] leads to reduction in the phosphorylation level of Mto2. This coincides with enhanced physical interaction between Mto1 and Mto2 and a significant increase in the number of Mto1 and Mto2 puncta, resulting in an increased number of cytoplasmic microtubules. Furthermore, during the transition from interphase to mitosis a *mor2-786* mutant shows aberrant mitotic cells containing both intra-nuclear short spindle microtubules (spindle length 1–4 µm) and cytoplasmic microtubules, resulting in attenuation of spindle microtubule assembly, posing a risk of chromosome segregation defects. On the other hand, forced association of Mor2 or Orb6, which are downstream components of the MOR, with Mto2 produces the opposite effect, promoting Mto2 phosphorylation and strongly inhibiting its function: the number of cytoplasmic microtubules was decreased, as was observed in *mto2Δ* cells. These data suggest that the MOR may directly regulate Mto2 and that Mto2 may be a substrate of Orb6, the most downstream kinase of the MOR.

### 3.1. Phosphorylation of Mto2 by the NDR family kinase Orb6

**Figure 6.**
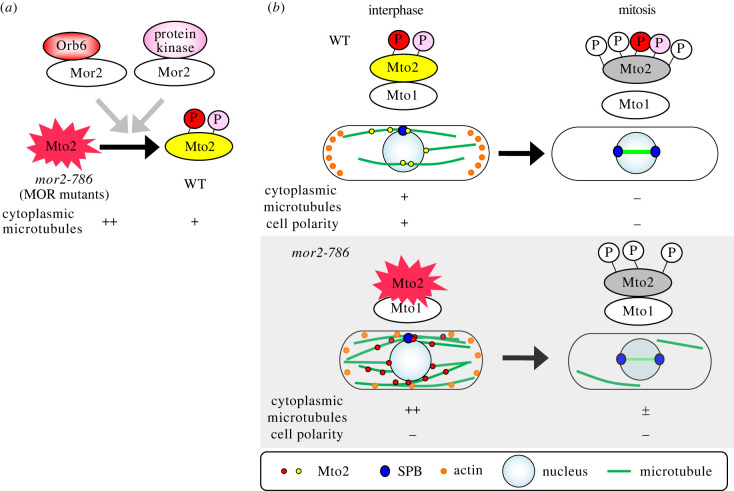
Model for cell cycle regulation of cytoplasmic microtubule organization by phosphorylation of Mto2 mediated by the MOR. (*a*) Orb6 and other protein kinases phosphorylate Mto2 through binding to Mor2. (*b*) Mto2 phosphorylation by Mor2 and Orb6 during interphase is crucial for reorganization of the microtubule cytoskeleton during the transition from interphase to mitosis.

Mto2 is phosphorylated on multiple sites during mitosis to inhibit its function and nucleation of cytoplasmic microtubules [14], suggesting that multiple kinases are likely to be involved in Mto2 phosphorylation during mitosis. However, Orb6 is unlikely to be the kinase responsible for this mitotic phosphorylation because Orb6 kinase is activated during interphase and then inactivated upon mitotic entry and maintained in an inactive state until completion of mitosis [16,19]. Orb6 may instead phosphorylate Mto2 during interphase. Indeed, Mto2 is also phosphorylated during interphase but to a lower level than it is in mitosis [14] and the MOR-dependent phosphorylation of Mto2 occurs when most *mor2-786* mutant cells start polarized growth after shift down to the permissive temperature from the restrictive temperature (figure 4*d*), suggesting that the MOR is involved in Mto2 phosphorylation during interphase, rather than mitosis, negatively regulating Mto2 for proper organization of cytoplasmic microtubules. In addition to Orb6 kinase, other protein kinases might phosphorylate Mto2 through interaction with Mor2 during interphase (figure 6*a*), as Mto2 was highly phosphorylated when Mor2 was forcibly bound to Mto2, compared to when Orb6 bound to Mto2 (figure 5*a*). Thus, Mor2 may function as a scaffold protein for phosphorylation of Mto2 by Orb6 and other protein kinases, but interaction between Mor2 and Mto2 is likely to be transient because forced association of Mor2 with Mto2 strongly inhibits Mto2 function: Mto2 is highly phosphorylated, compared to in the wild-type strain and the number of cytoplasmic microtubules is drastically reduced, as was observed in *mto2Δ* mutant cells (figure 5). A more detailed study of the molecular mechanisms will be important to uncover where and when Orb6 and other kinases regulate Mto2 through Mor2 during the cell cycle.

### 3.2. Roles of Mto2 phosphorylation by the MOR during interphase

What is the functional significance of Mto2 phosphorylation during interphase? The phosphorylation of Mto2 appears to control the number, distribution and activity of cytoplasmic MTOCs, and thereby affects cytoplasmic microtubule number and organization. Indeed, the number of Mto2 puncta and cytoplasmic microtubules in *mor2-786* mutant cells, in which the phosphorylation level of Mto2 is low (figure 4*b*), is increased approximately twofold, compared to that in wild-type cells (figures 2*a*–*c* and 3*a*). In addition, it is crucial to maintain an appropriate number of cytoplasmic microtubules during interphase for rapid and precise reorganization of the microtubule cytoskeleton during the transition from interphase to mitosis. Upon mitotic entry in wild-type cells, Mto2 is hyperphosphorylated and nucleation of cytoplasmic microtubules is ceased, but it takes a fraction of time to disassemble all cytoplasmic microtubules. Indeed, a substantial percentage of the mitotic cells with very short spindles (spindle length ≤1 µm; approx. prometaphase) contain interphase cytoplasmic microtubules, the disassembly of which is completed as the spindle formation progresses (spindle length 1–4 µm; approx. metaphase) [14]. On the other hand, in *mor2-786* mutant cells, in which cytoplasmic microtubule number is increased during interphase, 20% of the mitotic cells with short spindles (length 1–4 µm) still contain interphase cytoplasmic microtubules (figure 1*b*). Since spindle and cytoplasmic microtubules share same components, such as tubulin, the delay in reorganization of microtubule cytoskeletons observed in the *mor2-786* mutant strain affects spindle assembly and increases the risk of chromosome segregation defects. Indeed, the *mor2-786* mutant strain is sensitive to microtubule-depolymerizing drugs and the percentage of cells with some defect in chromosome segregation is increased in the presence of the drug, compared to that in the drug-treated wild-type strain (figure 1*d*,*e*). Furthermore, acceleration of the G2-M transition in the *mor2-786* mutant by combination with mutation of *wee1* [43], which is a negative regulator of CDK, showed more severe growth defects at semi-restrictive temperature than *mor2-786* single-mutant cells (electronic supplementary material, figure S8). Taken together, we conclude that phosphorylation of Mto2 by the MOR during interphase tunes its activity and underlies appropriate cytoplasmic microtubule organization, which is crucial for rapid and precise reorganization of microtubule cytoskeletons during the transition from interphase to mitosis (figure 6).

Our studies have shed light on novel functions of the MOR in non-centrosomal MTOCs and cytoplasmic microtubule organization. Mor2 and Orb6 proteins are evolutionarily conserved from yeast to humans [15–17,27,32] and in many cases, Mor2/Fry functions as an activator or a scaffold protein of Orb6/NDR kinase [32]. It has been reported that mammalian Fry localizes on spindle microtubules in mitosis and activates NDR1 kinase to achieve precise chromosome alignment [29,32]. Furthermore, mammalian Fry localizes to centrosomes and functions as a scaffold for Aurora kinase A-mediated Plk1 activation during mitosis and depletion of Fry causes centrosome and centriole splitting in mitosis resulting in the formation of multipolar spindles which are often seen in cancer cells [30,32]. Mammalian NDR kinase also regulates centrosome duplication [44] and a recent study revealed that NDR kinase functions downstream of the Hippo tumour suppressor pathway [45] which has key roles in regulation of centrosome number [46]. Thus, our findings reporting the role of the MOR in negative regulation of cytoplasmic MTOCs and further study using the tractable system of fission yeast could help to understand the detailed mechanisms controlling organization and reorganization of MTOCs and microtubule cytoskeletons during the cell cycle in other eukaryotic cells.

## 4. Methods

### 4.1. Yeast general methods

Standard media and methods for *S. pombe* were used [47]. Strains used in this study are listed in electronic supplementary material, table S1. Gene deletion and tagging were carried out by PCR and homologous recombination at the corresponding genomic loci [48,49].

### 4.2. Microscopy and image analysis

Cell morphology and nuclear division were observed by staining fixed cells with calcofluor and 4′,6-diamidino-2-phenylindole (DAPI), respectively, and were imaged with an Olympus epifluorescence microscope equipped with a video-micro-meter (Olympus), Olympus UVFL x40 (numerical aperture 1.3) and oil-immersion objective lens, DAPI filter sets (a 345 nm filter for excitation and a 445 nm filter for emission) illuminated with a 100 W HBO mercury short-arc lamp, and a CCD (charge-coupled device) camera (Ikegami), or with an Axioplan 2 microscope (ZEISS) equipped with Axiophoto (software), ZEISS PlanNEO-FLUARx100 (numerical aperture 1.3) and oil-immersion objective lens, DAPI filter sets (a 365 nm filter for excitation and a 445 nm filter for emission) illuminated with a 100 W HBO mercury short-arc lamp, and an AxioCam MRm CCD camera. Protein localization images were obtained using the DeltaVision RT system (Applied Precision) using an Olympus IX70 wide-field inverted fluorescence microscope with an Olympus PlanApo ×60 (numerical aperture 1.4) and oil-immersion objective lens and fluorescent filters (FITC: a 464–492 nm filter for excitation and a 500–523 nm filter for emission; TRITC: a 531–556 nm filter for excitation and a 564–611 nm filter for emission; and DAPI: a 381–401 nm filter for excitation and a 409–456 nm filter for emission) illuminated with a 100 W HBO mercury short-arc lamp. Cells were imaged in 12–14 Z-sections of 0.3 µm each. The images were captured by a CoolSNAP HQ camera (Roper Scientific) and processed by iterative constrained deconvolution using SoftWorx Version 3.5.1 (Applied Precision) and merged into a single projection with maximum intensity using ImageJ. For quantitative microtubule localization, cells expressing GFP-Atb2 were imaged in 12 Z-sections of 0.3 µm each, and the merged images were analysed in ImageJ. Fluorescence intensity of spindle microtubule (length is between 1 and 4 µm) over the background intensity was measured and used for statistical data analysis. For quantification of Mto1-GFP and Mto2-GFP puncta, cells expressing Mto1-GFP or Mto2-GFP were imaged in 14 Z-sections of 0.3 µm each, and the merged images were analysed in ImageJ. The number of puncta signals in single cells and the fluorescent intensity of the puncta over the background were measured. The SPB was identified using Sad1-dsRED as a SPB marker protein.

### 4.3. Immunochemistry

Preparation of cell extracts and immunoprecipitation were performed as follows. 5 × 10^8^ cells were collected by centrifugation. All subsequent manipulations were carried out at 4°C or on ice. Cells were broken in POM buffer (25 mM HEPES at pH7.4, containing 0.1% Triton X-100, 10% glycerol, 50 mM potassium acetate, 50 mM NaF, 60 mM β-glycerolphosphate, 2 mM EDTA, 1 mM dithiothreitol, 0.1 mM sodium vanadate, 15 mM *p*-nitrophenylphosphate, 40 µg ml^−1^ aprotinin, 20 µg ml^−1^ leupeptin, 1 µg ml^−1^ pepstatin and 1 mM phenylmethylsulfonyl fluoride) with glass beads by voltex. Extracts were cleared by centrifugation for 2 min at 7000 r.p.m. Protein concentrations were measured with a Bradford assay kit (Bio-Rad, Hercules, CA, USA). For immunoprecipitation of Mto1-FLAG, 6 mg of protein was pretreated with magnetic beads conjugated to protein G (Dynabeads, DYNAL, Thermo Fisher Scientific, Waltham, MA, USA) by incubation at 4°C with rotation for 1 h. The pretreated lysates were then separated from the beads and incubated with fresh protein G and a monoclonal anti-FLAG antibody (M2, Sigma-Aldrich, St Louis, MO, USA) at 4°C for 2 h. The beads were then washed in POM buffer, and cell extracts or precipitates were separated by SDS-PAGE and analysed by immunoblotting. λ-Phosphatase (λ-PPase, New England Biolabs, Inc., Beverly, MA, USA) was used for phosphatase treatment. Cell extracts were incubated at 30°C for 1 h in the presence or the absence of λ-PPase (400 units). Antibody probes used for immunoblotting were as follows: anti-GFP (Roche Holding AG, Basel, Switzerland), anti-FLAG (M2, Sigma-Aldrich, St Louis, MO, USA) and anti-α-tubulin (TAT-1, provided by K. Gull, Oxford University, UK) antibodies.

### 4.4. *In vitro* kinase assays

Full-length Mto2 was cloned into pJK148 vector and the Mto2 fragment was inserted into a pET28a vector with restriction enzymes (Sac1 and Xho1). His-tagged full length of Mto2 was expressed in BL21-CodonPlus-RP cells. Because His-Mto2 was insoluble, the inclusion bodies from the insoluble fraction were firstly purified with 8 M of urea (50 mM Tris, 1 mM EDTA, pH = 8.0). To reduce the urea concentration, the dialysis was carried out with 4 and 2 M of urea for 2 h each and with 1 M of urea overnight at 4°C. For *in vitro* kinase assays, the purified proteins were incubated with immunoprecipitated Orb6-GFP from wild-type or *mor2-786* mutant cells by using anti-GFP (Roche Holding AG, Basel, Switzerland) in kinase buffer with [*γ*^32^P] in 30 µl reactions at 30°C for 20 min. Reactions were stopped by adding sample buffer and boiling for 5 min. Reactions were separated by SDS-PAGE analysis. The incorporation of the radioactive phosphates was measured by autoradiography and substrate proteins were stained with Coomassie Brilliant Blue (CBB).

### 4.5. Statistics and reproducibility

All experiments were independently conducted at least three times. The individual data are shown with means and s.d. or s.e.m. *p*-value was determined either by two-tailed unpaired Student's *t*-test or a two-sample *t*-test. *p*-values are shown in the legends.

## Data Availability

The data are provided in electronic supplementary material [50].
